# Case Report: An Unusual Case of Acute Lower Limb Ischemia as Precursor of the Asherson's Syndrome

**DOI:** 10.3389/fcvm.2021.727276

**Published:** 2021-10-21

**Authors:** Edoardo Pasqui, Silvia Camarri, Gianmarco de Donato, Stefano Gonnelli, Giancarlo Palasciano, Luigi Gennari

**Affiliations:** ^1^Department of Vascular Surgery, University of Siena, Siena, Italy; ^2^Department of Medicine, Surgery and Neurosciences, University of Siena, Siena, Italy

**Keywords:** acute limb ischemia, Antiphospholipid Syndrome, Catastrophic Antiphospholipid Syndrome, autoimmune disease, peripheral artery disease

## Abstract

**Introduction:** Asherson's Syndrome, also defined as Catastrophic Antiphospholipid Syndrome (CAPS), represents the most severe manifestation of Antiphospholipid Antibody Syndrome. Rarely, the first CAPS diagnosis is based on macro-thrombotic event as acute limb ischemia.

**Case Presentation:** We present a case of a 65-year-old woman admitted with an acute lower limb arterial ischemia with a complete occlusion of all the three tibial vessels. Three endovascular recanalization procedures were performed contemporary to 48 h intraarterial thrombolysis administration. The patency of tibial arteries was restored with a near-complete absence of digital arteries and microvessel perfusion of the foot. In the following days, an aggressive foot gangrene was established, leading to a major lower-limb amputation. Due to the general clinical status worsening and aggressiveness of ischemic condition, further investigations were performed leading to the diagnosis of an aggressive Asherson's Syndrome that was also complicated by a severe heparin-induced thrombocytopenia. Medical management with a high dose of intravenous steroids and nine sessions of plasma exchange led to a clinical condition stabilization.

**Conclusion:** In our case, the presence of a “sine causa” acute arterial occlusion of a large vessel represented the first manifestation of an aggressive form of Asherson's Syndrome that could represent a fatal disease. Due to the extreme variety of manifestations, early clinical suspicion, diagnosis, and multidisciplinary management are essential to limit the life-threatening consequences of patients.

## Keypoints

1. Catastrophic Antiphospholipid Syndrome (CAPS) is the most severe manifestation of Antiphospholipid Antibody Syndrome.
2. Acute limb ischemia is one of the less common presentations of CAPS.
3. The case presented is an aggressive CAPS manifestation complicated by a severe heparin-induced thrombocytopenia that also led to a major lower-limb amputation.
4. CAPS is a life-threatening condition that needs a multidisciplinary management in order to save the lives of patients.

## Introduction

Asherson's Syndrome, also defined as Catastrophic Antiphospholipid Syndrome (CAPS), represents the most severe manifestation of Antiphospholipid Syndrome (APS). The incidence of CAPS is <1% of the APS cases (1). It is often associated with a high rate of mortality (>50%), usually as a consequence of multiorgan failure (2). Rarely, the first manifestation of CAPS is a macro-thrombotic event as acute limb ischemia (ALI). This paper shows a case of a woman admitted with an ALI as the first symptom of an aggressive Asherson's Syndrome complicated by a severe heparin-induced thrombocytopenia (HIT).

## Case Report

We present a case of a 65-year-old woman who was admitted to the Emergency Department with left lower limb pain that had occurred 10 days before. The patient was an active smoker and her gynecological history revealed three abortions during the first trimester out of four pregnancies. No previous thrombotic/embolic events were reported. The clinical vascular evaluation highlighted clear signs of foot malperfusion, absence of tibial pulses, and cyanosis (Rutherford classification for ALI: IIb). Duplex ultrasound examination confirmed the complete occlusion of all tibial vessels with a good patency of superficial femoral artery and popliteal artery. Lower limb arterial tree was not characterized by substantial chronic atherosclerotic lesions, and electrocardiogram confirmed the absence of cardiac arrhythmias.

Analgesic drugs and intravenous unfractioned heparin were promptly administered. Table 1 shows laboratory findings at admission. In the following hours, she was moved to the operating theater to undergo an urgent endovascular clot removal. Standard surgical embolectomy was not chosen as first-line approach due to the distal and diffuse localization of the arterial occlusion difficult to treat with Fogarty's embolectomy balloon catheter. The intraoperative angiography confirmed the total occlusion of the distal portion of the anterior (ATA), posterior (PTA), and peroneal tibial arteries with complete absence of distal perfusion at the level of the foot.

**Table 1:** Laboratory tests during hospitalization.

**Laboratory tests**	**At admission**	**The day after amputation**	**5th day after amputation**
Hemoglobin (g/dl)	11.1	9.3	10.8
White blood count (1000/μL)	15.77	11.9	8.51
Neutrophyl (1000/μL)	13.43	9.83	5.26
Lymphocyte (1000/μL)	1	1.21	1.63
Platelets (1000/μL)	228	329	1
INR	1.61		1.54
PTT (s)	92		134
Creatinine (mg/dl)	0.54	1.03	2.4
C reactive protein (mg/dl)	15.82	19.78	9.75
Creatine kinase (UI/L)	3311	1241	3
HS-Troponin (ng/l)	N/A	2907	N/A

^*^INR, International Normalized Ratio; PTT, Partial Thromboplastin Time; HS-Troponin, High Sensibility Troponin.

**Figure 1:**
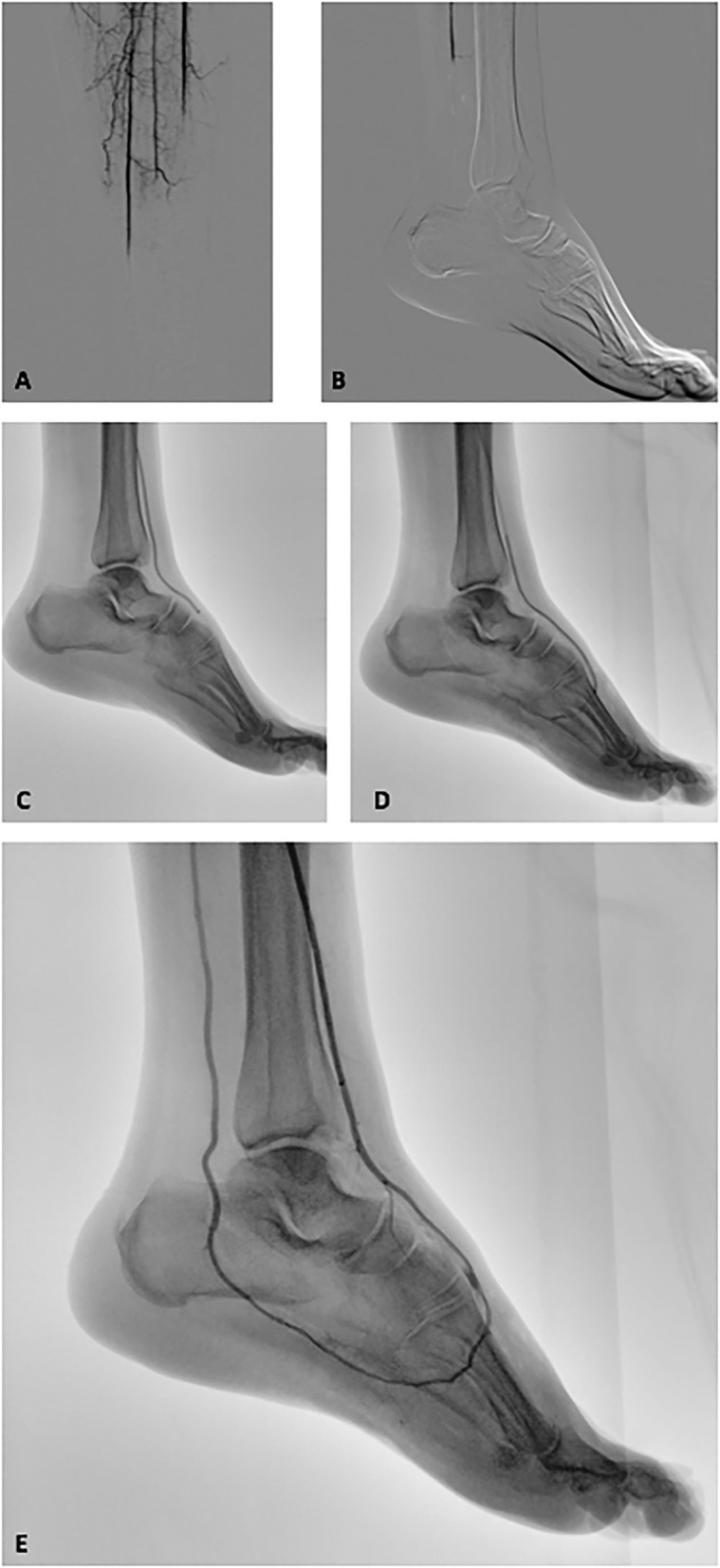
Intraoperative fluoroscopic images of the revascularization procedure of the ischemic lower limb. **(A,B)** Intraprocedural angiography revealed a below-the-knee complete occlusion on all the tibial vessels and a complete absence of distal foot perfusion. **(C)** Penumbra Indigo thromboaspiration catheter CAT 6 advanced in the anterior tibial artery and dorsalis pedis. **(D)** Thromboaspiration catheter CAT 6 advanced into the plantar arch. **(E)** Post-thromboaspiration angiography revealed the complete recanalization of the anterior tibial artery, dorsal, and plantar arch and posterior tibial artery. A near-complete absence of interdigital arteries and distal vasculature opacization is also highlighted.

Endovascular catheter thromboaspiration maneuvers with Indigo CAT 6 + SEP 6 (Penumbra Inc, Alameda, CA, USA) system were performed with removal of organized thrombotic material. The recanalization procedure achieved a good result, reaching the more distal part of the foot arterial tree using the plantar loop technique (3) (Figure 1). Although that maneuver was successful to reestablish the flow at level of plantar arch, no flow was appreciated into digital arteries. In order to achieve the complete resolution of the ischemia, the surgical team decided to start a 24-h local catheter-directed thrombolysis (CDT) with recombinant Tissue-Plasminogen-Activator. A 24- and 48-h angiography revealed the same situation of severe delay of the contrast medium washout at below-the-ankle vessels with near-complete absence of digital arteries and microvessel perfusion of the foot (Figure 1).

Postoperative days were characterized by a worsening of the general clinical status. An aggressive ischemic foot gangrene developed contemporary to a gradual increase of inflammatory markers, fever onset, and neutrophil increase suggesting a septic condition due to the foot and leg gangrene. Twelve days after admission, a below-the-knee amputation was mandatory to limit the progression of ischemic gangrene.

In the next 2 days, the patient manifested a temporary general clinical improvement, followed by a novel general clinical worsening with altered mental status (drowsiness, headache, and hallucinations), acute kidney injury, and acute coronary syndrome (non-ST segment elevation myocardial infarction) and very severe thrombocytopenia.

Because of the unclear arterial occlusion etiology, the anamnestic features, and rapid clinical deterioration, a full panel of oncological and autoimmune disease markers was requested.

Oncological markers were negative, but autoimmune screening reported a scenario compatible with triple-positive APS (Lupus Anticoagulant, Anti-β2GPI, Anticardiolipin Immunoglobulin) as shown in Table 2.

**Table 2:** Autoimmune and coagulation panels tested on the day of Asherson's Syndrome Diagnosis.

**Autoimmune and coagulation panels**	**Patient's result**	**Reference level**
ANA	+	N/A
Anticardiolipin Ab (U/ml)		
IGG	1150	N/A
IGM	124.4	
Anti-β2GPI Ab (U/ml)		
IGG	>6100	N/A
IGM	242	
LAC ratio	5.9	N/A
Anti PLT Ab heparin-induced	+	N/A
D-dimer (ng/mL FEU)	3880	<500
Quick (%)	54	80–120
INR	1.59	0.8–1.2
PTT (s)	134	21–37
Antithromin III (%)	57	70–120
Factor II (%)	62	72–120
Factor V (%)	109	70–120
Factor VII (%)	51	60–160
Factor VIII (%)	28.4	55–150
Factor IX (%)	2	60–130
Factor X (%)	62	70–120
Factor XI (%)	2	65–150
Factor XII (%)	2	60–150
C protein (%)	54	70–146
Protein S (%)	59	60%
Thrombin/Antithrombin ratio (micrg/l)	5	1–4.1
Antithrombin III (%)	57	N/A
PT mix ratio	1.33	N/A
PTT mix ratio	3.46	N/A

^*^ANA, Antinuclear Antibodies; Ab, Antibodies; LAC, Lupus Anticoagulant; INR, International Normalized Ratio; PTT, Partial Thromboplastin Time; PT, Prothrombin Time.

In this perspective, a hematological counseling was requested and, based on clinical and laboratoristic data, the life-threatening CAPS diagnosis was confirmed.

Due to the extreme severity of thrombocytopenia, further investigations were performed, and the positiveness of anti-PF4/heparin emerged defining the diagnosis of a contemporary HIT.

A high dose of intravenous steroids (2 mg/kg/die methylprednisolone) was promptly administered. Moreover, nine sessions of plasma exchange were performed due to the extremely critical and unresponsive condition of the patient. Intravenous immunoglobulins were not chosen because of the renal impairment (plasmatic creatinine 2.5 mg/l). This aggressive treatment determined a slow but constant improvement of laboratory tests and clinical status.

Two days after the final session of plasma exchange, platelets count reached a value of over 70.000/mm^3^ and anticoagulant therapy was restarted with Fondaparinux. Forty-one days after the admission, the patient was discharged in good general clinical condition.

The patient followed a strict clinical and instrumental follow-up. Anticoagulation therapy with vitamin K antagonist was started in order to reduce the recurrence of thromboembolic events. In addition, low-dose of corticosteroids was continued.

After 3 months from the amputation, it was possible to undertake the multidisciplinary path for the leg prosthesis configuration and production reaching a good quality of life.

## Discussions

Antiphospholipid Syndrome represents a systemic autoimmune disease, characterized by both thrombotic and non-thrombotic manifestations. CAPS is the most severe condition of APS with multi-organ involvement related to typically diffuse micro-thrombotic events (4). Clinical manifestation is characterized by a gradual and progressive systemic involvement. Half of the cases involve the lungs, central nervous system, heart, kidney, liver, skin, and gastrointestinal tract. Atypical manifestations involve the adrenal, pancreatic, spleen, and testicular vessels. The case presented offers a clear example of this life-threatening condition with significant acute kidney failure, neurological impairment, non-ST elevation myocardial infarction with no significant consequences on cardiac function, and a significant increase of liver cytonecrosis markers and hypoalbuminemia. The pathophysiological mechanism remains unclear. Kitchens et al. postulated that vascular occlusion itself triggers additional thrombosis, introducing the concept of “thrombotic storm” (5). He proposed that clots continue to generate thrombin, fibrinolysis is depressed by an increase in plasminogen activator inhibitor type 1, and there is a consumption of the natural anticoagulant proteins such as protein C and antithrombin determining a “vicious cycle.” Further studies outlined the triggering role of antiphospholipid antibodies in activating endothelial cells and establishing the main pathological pathway (6) commonly denominated thrombosis mediated by antibodies (7).

Based on the thrombotic storm concept, numerous comparable disorders with an extreme prothrombotic presentation may share a similar underlying pathophysiologic process representing an extreme response to an initial prothrombotic stimulus. Thus, the genetics of thrombotic storm are currently being investigated with the hypothesis that prothrombotic genetic risk factors trigger an accelerated form of thrombosis following an initial event.

CAPS occurrence is also strongly related with pregnancy, puerperium (8) representing almost 6% of all cases. This represents a life-threatening situation with a high mortality rate in these young women of childbearing age. This also represents a unique scenario where many factors may participate as additional potential trigger factors, including infections such as endometritis, cesarean wound or episiotomy wound infection or mastitis, lupus flares, anticoagulation withdrawal during the actual labor, among others.

Oral contraceptives assumption could also be an additive risk factor for CAPS occurrence (9), as presented by McRae and colleagues in their recent paper.

The contemporary presence of HIT, which concurred after the administration of unfractioned heparin for ALI management, increased the complexity of the setting needing of the patient for even more strict attention.

We think that the patient arrived at our attention with an already established APS exacerbation; the progression of foot gangrene and the consecutive amputation determined the final trigger for the CAPS aggressive onset moving the patients toward a life-threatening condition.

In our case, the presence of a “sine causa” acute arterial occlusion of a large vessel represented the first manifestation of an aggressive form of Asherson's Syndrome that could represent a fatal disease. Due to the extreme variety of manifestations, early clinical suspicion, diagnosis, and multidisciplinary management are essential to limit the life-threatening consequences of patients.

## Patient Perspective

Antiphospholipid Syndrome represents an underestimated autoimmune condition, majorly involving female patients. In this perspective, the early diagnosis and management could reduce significant thromboembolic events that may lead to life-threatening events. The most aggressive clinical manifestation of APS, known as Asherson's Syndrome, has to be promptly recognized especially in female patients presenting with unclear arterial and/or venous thromboembolic events, as presented in this paper. Even if this condition is quite uncommon, the consequences of a delayed management could be devastating. From this assumption, patients and physicians have to be aware of the aggressiveness of this scenario, especially when the etiology of the index thromboembolic event remains uncertain. In these cases, autoimmune screening at admission could reduce life- and limb-threatening conditions.

The patient herself lived this devastating experience with great anxiety; she reports that she does not remember much of her hospital stay, especially from the moment of the worsening of her clinical status (immediately after the amputation) until the clinical recovery occurred during the numerous cycles of plasmapheresis. The loss of her leg was accepted, albeit with difficulty, with the help of a psychologist who allowed her to accept the condition of this new life.

## Data Availability Statement

The original contributions presented in the study are included in the article/supplementary material, further inquiries can be directed to the corresponding author.

## Ethics Statement

Written informed consent was obtained from the relevant individual for the publication of any potentially identifiable images or data included in this article.

## Author Contributions

All authors listed have made a substantial, direct and intellectual contribution to the work, and approved it for publication.

## Conflict of Interest

The authors declare that the research was conducted in the absence of any commercial or financial relationships that could be construed as a potential conflict of interest.

## Publisher's Note

All claims expressed in this article are solely those of the authors and do not necessarily represent those of their affiliated organizations, or those of the publisher, the editors and the reviewers. Any product that may be evaluated in this article, or claim that may be made by its manufacturer, is not guaranteed or endorsed by the publisher.

## Abbreviations

CAPS: Catastrophic Antiphospholipid Syndrome
APS: Antiphospholipid Syndrome
ALI: Acute Limb Ischemia
CDT: Catheter Directed Thrombolysis
HIT: Heparin-Induced Thrombocytopenia.
